# Bleeding events in thrombotic antiphospholipid syndrome: prevalence, severity, and associated damage accrual

**DOI:** 10.1016/j.rpth.2024.102327

**Published:** 2024-01-26

**Authors:** Pedro Gaspar, Prabal Mittal, Hannah Cohen, David A. Isenberg

**Affiliations:** 1 Department of Internal Medicine, Hospital Santa Maria, Unidade Local de Saúde Santa Maria, Lisbon, Portugal; 2 Instituto de Medicina Molecular João Lobo Antunes, Centro Académico de Medicina de Lisboa, Faculdade de Medicina, Universidade de Lisboa, Lisbon, Portugal; 3 Department of Haematology, University College London Hospitals NHS Foundation Trust, London, UK; 4 Haemostasis Research Unit, Department of Haematology, University College London, London, UK; 5 Centre for Rheumatology, Division of Medicine, University College London, London, UK

**Keywords:** anticoagulants, antiphospholipid syndrome, antithrombotic agents, bleeding, damage, prevalence

## Abstract

**Background:**

Life-long anticoagulation increases bleeding risk in patients with antiphospholipid syndrome (APS). The Damage Index for Antiphospholipid Syndrome does not include bleeding events in damage accrual.

**Objectives:**

We aimed to characterize the prevalence, severity, and damage associated with bleeding events in patients with APS.

**Methods:**

This was a single-center retrospective analysis of patients with thrombotic APS (2006 Sydney criteria). Bleeding events were reviewed up to 43 years and classified according to the ISTH definitions into 2 groups: 1) major bleedings and 2) nonmajor bleedings (minor bleedings and clinically relevant nonmajor bleedings). Damage events were recorded as bleeding events a) resulting in permanent (>6 months) decrease in organ function and b) complicated by total/partial organ resection.

**Results:**

Among 197 patients (2412 patient-years [PYs] of follow-up), all of whom had been exposed to antithrombotic therapy, 40.6% experienced 167 bleedings (6.9 events per 100 PYs), of whom 61.3% had nonmajor bleedings (77.2% of bleedings: 42.6% minor, 57.4% clinically relevant nonmajor) and 38.8% had major bleedings (22.8% of bleedings; 1.6 events per 100 PYs). Soft/connective tissue was affected in 44.3% of bleedings, and 94.6% were nonmajor bleedings. Central nervous system was affected in 20.9% of bleedings, and 62.9% were major bleedings. Bleeding events were spontaneous in 90.4% of cases, and thrombocytopenia was likely involved in 62.2% of bleedings. Damage occurred in 11.4% of bleedings and affected 7.6% of patients. Most of the damage was associated with central nervous system events (8.4% of all bleedings).

**Conclusion:**

Approximately 40% of patients experienced at least 1 bleeding, and almost 8% of patients were left with organ damage not recognized by the current version of the Damage Index for Antiphospholipid Syndrome.

## Essentials

• The Damage Index for Antiphospholipid Syndrome does not include bleedings as damage.
• We analyzed all bleedings occurring in a group of 197 patients with antiphospholipid syndrome.
• For every 100 patients followed in 1 year, 1.6 experienced at least 1 major bleeding.
• Damage arose in ∼8% of all patients, mainly in the central nervous system.

## 1 Introduction

Thrombotic antiphospholipid syndrome (APS) is a multisystemic autoimmune acquired thrombophilia causing venous, arterial, and/or small vessel thrombotic events [1]. Standard of care relies on vitamin-K antagonists (VKAs) [2], which are associated with an increased risk of bleeding complications [3]. Nevertheless, bleeding events in APS are poorly reported in the literature and their classification remains arbitrary based on clinical judgment in most studies [[4], [5], [6]]. During the 10-year follow-up period of the Euro-Phospholipid project including 1000 patients with APS, 10.7% of all deaths were attributed to bleeding [4]. The authors reported 61 major hemorrhages, but no specification is given regarding the exact number of affected patients (ie, a patient can suffer from >1 bleeding event) [4]. In a small single-center retrospective study reviewing 35 patients with APS followed for a median of 20 years, bleeding events were present in 34% of patients and were classified as severe in 8% (*n* = 3) of patients [5]. More recently, Serrano et al. [6] showed that 15 severe bleedings occurred in 15 out of the 160 patients (9.4%) who were followed prospectively. Importantly, no objective and reproducible classification score was used to classify bleeding events as severe in these studies.

Significant advances have been made in how we assess damage in patients with rheumatic diseases. The Systemic Lupus International Collaborating Clinics group agreed that damage might be the consequence of disease activity, treatment, or concomitant disease and this is reflected in the Systemic Lupus International Collaborating Clinics/American College of Rheumatology Damage Index [7], which includes cataracts, osteoporosis, and diabetes as damage, which are recognized as consequences of long-term exposure to steroids. Bone marrow failure and chronic chemical cystitis secondary to cyclophosphamide use are considered in the vasculitis damage index [8].

Despite adequate treatment, patients with APS experience recurrent thrombotic events that lead to organ damage and carry increased risk of morbidity and mortality [9,10]. The Damage Index for Antiphospholipid Syndrome (DIAPS) is the only validated damage score for thrombotic APS reported to date [11]. The DIAPS is a 38-item score that was designed to capture the thrombotic APS-specific features, but unlike other rheumatic disease–specific damage indexes, it does not include drug-related damage [11,12].

Our aim was to describe the cumulative prevalence and severity of bleeding events and its related damage in a group of patients with thrombotic APS followed for >30 years.

## 2 Methods

### 2.1 Patients and data collection

In this study, we retrospectively analyzed, in a service evaluation, a group of 197 patients with thrombotic APS attending the rheumatology and/or hematology clinics at the University College of London Hospital until December 2019. We reviewed patient files for up to 43 years. Both departments have dedicated APS clinics where patients have been followed prospectively since the early 80s. We included patients with thrombotic primary APS as well as APS secondary to other autoimmune condition. Patients with purely obstetrical APS and those aged <18 years were not included in this analysis. During the long follow-up period, antiphospholipid antibody determination followed classification criteria at that specific time. Nevertheless, all patients included here met the 2006 Sydney classification criteria [1]. All clinical and laboratory data were obtained through the detailed analysis of all paper files and electronic records, and all information is reported whenever available. Data were collected by 2 physicians who are actively involved in the clinical follow-up of patients with APS. The senior authors were consulted whenever doubt existed. Information collected into the protocol forms was transferred to an anonymized computerized database.

### 2.2 Definitions

A protocol form was used to record the clinical and serologic characteristics of the patients. Namely, it included the following: 1) sex, 2) race, 3) age at disease onset (defined as the first thrombotic event related to APS), 4) current age (defined as age at study entry), 5) type of APS and associated underlying autoimmune disease, 6) clinical manifestation at disease onset, 7) cumulative clinical manifestations (until entry in the study), 8) treatment regimen and therapeutic targets (international normalized ratio [INR] value; time in therapeutic INR value), 9) accrual damage (accessed using the DIAPS [11]), 10) bleeding events, and 11) laboratory features.

Clinical manifestations were taken as recorded in medical notes and confirmed according to established criteria for each manifestation using all available laboratory, imaging, and/or histopathologic studies. At our institution, patients are requested to self-nominate the ethnic group they belong to. We have thus used the categories recorded in the hospital notes. The treatment regimen was taken as ever-present (ie, exposure to anticoagulation and/or antiplatelet therapy). We have also recorded data regarding the following: 1) the latest treatment modality (at entry into the protocol study), 2) the latest treatment intensity (ie, INR range value; low-molecular-dose heparin and direct anticoagulant dose), and 3) the treatment regimen and its intensity around each bleeding event (precision period of ±1 month). Thrombocytopenia (<150 × 10^9^ platelets/L) was classified as mild (100-150 × 10^9^ platelets/L), moderate (50-100 × 10^9^ platelets/L), and severe (<50 × 10^9^ platelets/L) and recorded as the lowest value ever-present. When the absolute values were not found, thrombocytopenia was taken as stated in the medical records and/or classified by clinical judgment when there was little difficulty assigning a category (eg, immune thrombocytopenia requiring splenectomy—classified as severe). When present, thrombocytopenia was classified as “likely involved in bleeding” if it occurred near the bleeding event (precision period of ±1 month).

Bleeding events were classified according to the ISTH definitions for minor bleedings [13], clinically relevant nonmajor (CRNM) bleedings [13], and major bleedings in nonsurgical [14] and surgical [15] patients. Distinguishing a minor bleeding event from a CRNM bleeding could be very challenging in retrospective studies, especially when some notes date >30 years ago. For example, epistaxis or an increase in menstrual flow after starting anticoagulation would not be classified as CRNM bleeding unless it prompted a face-to-face evaluation for a physical examination or laboratory testing. Whenever doubt existed, we classified the bleeding event as a minor event. To help overcome this classification issue and make the results more readily applicable in clinical practice, the patients were further classified into 2 main groups: 1) major bleeding events, which included major bleeding events in nonsurgical and surgical settings, regardless of whether these patients had also suffered minor and/or CRNM bleedings, and 2) nonmajor bleeding events, including patients who only experienced minor and/or CRNM bleeding events. Only bleeding events that occurred after the diagnosis of APS and during treatment with antithrombotic therapy (antiplatelet and/or anticoagulation) were considered. The bleeding event diagnosis and clinical characteristics were taken as recorded in medical notes and confirmed using all available laboratory and/or imaging studies. Attention was made on classifying a bleeding event as spontaneous or not (ie, resulting from trauma or any medical intervention such as surgery). As there was no definition of damage arising from bleedings in this population, we considered damage every time a bleeding event, regardless of its classification, resulted in a permanent decrease (lasting for at least 6 months) in organ function according to the definition of the DIAPS [11] and/or resulted in organ resection.

### 2.3 Statistical analysis

The descriptive analysis was performed using STATA version 16. Data are shown as number (%) for categorical variables and median (IQR) for continuous variables. The annualized incidence rate was calculated for all bleeding events and for major bleeding events. We handled missing data using a list-wise deletion approach; we dropped from a specific analysis any patient who had a missing value in that specific variable (ie, number/[total number − dropped values]). Failure curves were obtained using the Kaplan-Meier method for all bleeding events and for major bleeding events.

## 3 Results

### 3.1 Clinical description and bleeding event characterization

Patients’ demographic and clinical characteristics and the main outcomes are summarized in Table 1, Table 2. We identified 197 patients with thrombotic APS, most being females (71.1%) with primary APS (65.9%). The median age at APS onset was 40 years (IQR: 28-51 years), and patients were followed for a median of 10 years (IQR: 6-17 years; 2412 patients-years). Venous thromboembolism was the most frequent initial thrombotic manifestation (58.8%), followed by arterial (44.2%) and microvascular (2.5%) thromboses. We identified 438 cumulative thrombotic events in our population, with the most frequent being lower limb deep venous thrombosis (26.7%), pulmonary embolism, and ischemic stroke (both 19.9%). All patients were exposed to anticoagulation and/or antiplatelet therapy at some point during follow-up. Almost all patients were treated with anticoagulation (98.5%), and 24.9% received both anticoagulation and antiplatelet therapy.

**Table 1:** Demographic and clinical characterization.

Total	197 (100.0)
Female sex, *n* (%)	140 (71.1)
Type of APS, *n* (%)	
Primary	130 (65.9)
Secondary	67 (34.0)
Systemic lupus erythematosus	57 (28.9)
Other^a^	10 (5.1)
Age at APS onset (y)	40 (28-51)
Time of follow-up (y)	10 (6-17)
Race, *n* (%)	
White	139/192 (72.4)
Black	18/192 (9.4)
Asian	19/192 (9.9)
Other	18/192 (9.4)
Type of first event, *n* (%)	
Venous	116 (58.8)
Arterial	87 (44.2)
Microvascular^b^	5 (2.5)
aPL profile (ever)	
LA	156 (79.2)
aCL (IgG and/or IgM)	116 (58.4)
aβ2GPI (IgG and/or IgM)	103 (52.3)
Triple aPL-positive	65 (32.9)
Double aPL-positive	48 (24.4)
LA + aCL (IgG and/or IgM)	25 (12.7)
LA + aβ2GPI (IgG and/or IgM)	10 (5.1)
aCL + aβ2GPI (both IgG and/or IgM)	13 (6.6)
Mono aPL-positive	84 (42.6)
LA	56 (28.4)
aCL (IgG and/or IgM)	13 (6.6)
aβ2GPI (IgG and/or IgM)	15 (7.6)
Treatment regimen (ever), *n* (%)	
Anticoagulation and/or antiplatelet therapy	197 (100.0)
Anticoagulation	194 (98.5)
Antiplatelet therapy	52 (26.4)
Anticoagulation and antiplatelet therapy	49 (24.9)
Thrombotic manifestations (cumulative), *n* (%)	438 (100.0)
Deep venous thrombosis	117 (26.7)
Lower limb	103 (23.5)
Upper limb	14 (3.2)
Pulmonary embolism	87 (19.9)
Ischemic stroke	87 (19.9)
Transient ischemic attack	43 (9.8)
Central venous sinus thrombosis	15 (3.4)
Peripheral arterial thrombosis	13 (3.0)
Upper limb	7 (1.6)
Lower limb	6 (1.4)
Myocardial infarction	12 (2.7)
Superficial venous thrombosis	7 (1.6)
Digit ischemia	6 (1.4)
Other central venous thrombosis^c^	5 (1.1)
Renal thrombotic microangiopathy	5 (1.1)
Other^d^	19 (4.3)

Data are shown as number (%) for categorical variables and median (IQR) for continuous variables. The denominators are provided if they differed from the overall numbers within the group.

aβ2GPI, anti–β2-glycopritein I antibodies; aCL, anticardiolipin antibodies; aPL, antiphospholipid antibodies; APS, antiphospholipid syndrome; DIAPS, Damage Index for Antiphospholipid Syndrome; LA, lupus anticoagulant.

a Other include the following: rheumatoid arthritis (*n* = 3, 1.5%), undifferentiated autoimmune rheumatic disease (*n* = 5, 2.5%), psoriatic arthritis (*n* = 1, 0.5%), and rhupus (*n* = 1, 0.5%).

b Biopsy proven.

c Internal jugular vein; vena cava.

d Other thrombotic manifestations include the following: splenic infarct (*n* = 4, 0.9%), chronic cutaneous ulcer (*n* = 3, 0.7%), adrenal insufficiency (*n* = 3, 0.7%), kidney infarct (*n* = 3, 0.7%), renal vein thrombosis (*n* = 2, 0.5%), mesenteric thrombosis (*n* = 2, 0.5%), and retina artery thrombosis (*n* = 2, 0.5%).

**Table 2:** Prevalence of bleeding events per decade and other outcomes.

Total	197 (100.0)
Patients diagnosed with bleeding events, *n* (%)^a^	80 (40.6)
1976-1989	0
Major bleedings	0
Nonmajor bleedings	0
1990-1999	5 (6.3)
Major bleedings	4 (5.0)
Nonmajor bleedings	1 (1.3)
2000-2009	24 (25.0)
Major bleedings	11 (13.8)
Nonmajor bleedings	9 (11.3)
2010-2019	62 (77.5)
Major bleedings	21 (26.3)
Nonmajor bleedings	41 (51.3)
Unknown^b^	10 (12.5)
Major bleedings	0
Nonmajor bleedings	10 (12.5)
Final DIAPS	
Median (IQR)	1 (0-2)
Mean ± SD	1.6 ± 1.7
Death	23 (11.7)
Time to death (y)	10 (6-17)

Data are shown as number (%) for categorical variables and median (IQR) for continuous variables.

DIAPS, Damage Index for Antiphospholipid Syndrome.

a The number of affected patients within each decade may vary from the overall total presented in Table 3, Table 4 as a patient may experience >1 bleeding event in different years (see Supplementary Tables S2 and S3 for further details).

b Not specified in medical notes.

**Figure 1:**
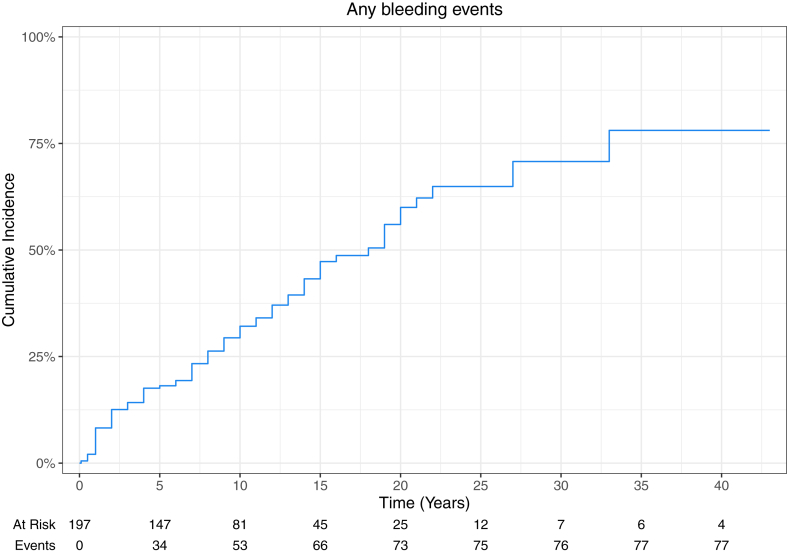
Kaplan-Meier failure curve showing cumulative incidence of all bleeding events over time. It was not possible to determine the date when 3 bleeding events occurred in 3 patients (Supplementary Table S2); hence, the total number of events (*n* = 77) differs from the total number of patients affected by any bleeding event (*n* = 80; Table 3).

**Figure 2:**
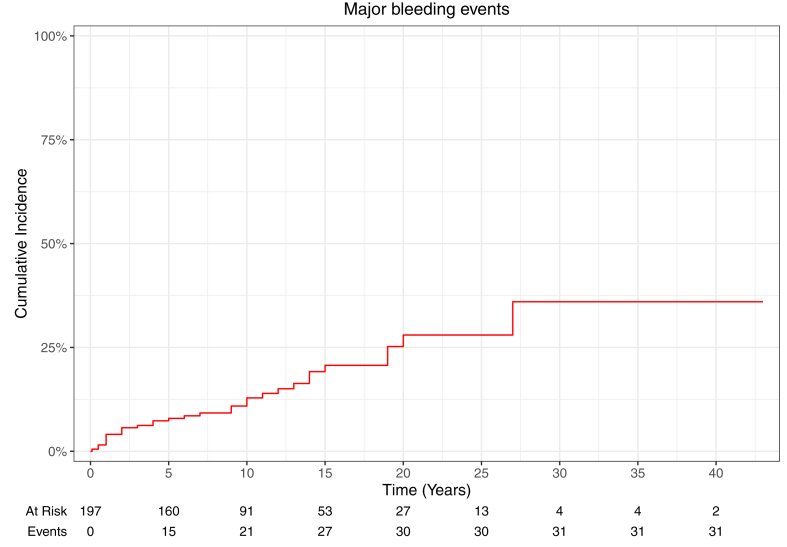
Kaplan-Meier failure curve showing cumulative incidence of major bleeding events over time.

During the study period, 40.6% (80/197) of patients experienced 167 bleeding events (Table 3). Supplementary Table S1 details the main clinical and demographic characteristics as well as the prevalence of any bleeding event and major bleeding event organized by time of follow-up. The incidence rate for any hemorrhagic complication was 6.9 events per 100 person-years, and it was 1.6 events per 100 person-years for major bleeding events. Figure 1 shows the cumulative incidence of all bleeding events over time. Regardless of the severity of the bleeding manifestation, soft/connective tissue were the most affected (44.3%), followed by the genitourinary (GU) system (22.2%), central nervous system (CNS; 20.9%), and abdominal compartment (10.8%). Bleeding events were classified as nonmajor bleeding events (minor bleedings: 42.6% [55/129]; CRNM bleedings: 57.4% [74/129]; calculated from Supplementary Tables S2 and S3) in 77.2% (129/167) of cases and affected 24.9% (49/197) of all patients (61.3% [49/80] of patients experiencing bleeding events). These events occurred more frequently in the soft/connective tissue (54.3% [70/129]) and GU systems (25.6% [33/129]), with easy bruising/soft tissue hematoma (15.5% [20/129]) and abnormal uterine bleeding (17.1% [22/129]) being the most prevalent manifestations. Bleeding events were classified as major bleeding events in 22.8% (38/167) of cases, affecting 15.7% (31/197) of all patients (38.8% [31/80] of patients experiencing bleeding events). Figure 2 shows the cumulative incidence of major bleeding events over time. Within this group, 57.9% (22/38) of major bleeding events occurred in the CNS, equally affecting the brain/spinal cord and the meninges (23.7% [9/38] of major bleeding events). The abdominal compartment was affected in 21.1% (8/38) of cases.

**Table 3:** Distribution of bleeding events and associated damage.

Events, *n* (%; %^a^)	Total bleeding events 167 (100.0)	Nonmajor bleedings 129 (77.2; 100.0)	Major bleedings 38 (22.8; 100.0)	Damage 19 (11.4; 100.0)
Soft/connective tissue, *n* (%; %^a^)	74 (44.3)	70 (41.9; 54.3)	4 (2.4; 10.5)	0
Easy bruising	15 (9.0)	15 (9.0; 11.6)	0	0
Soft tissue hematoma	6 (3.6)	5 (3.0; 3.9)	1 (0.6; 2.6)	0
Gum bleeding	6 (3.6)	6 (3.6; 4.7)	0	0
Epistaxis	16 (9.6)	16 (9.6; 12.4)	0	0
Hemoptysis	18 (10.8)	18 (10.8; 14)	0	0
Muscular hematoma	11 (6.6)	10 (6.0; 7.6)	1 (0.6; 2.6)	0
Hemarthrosis	2 (1.2)	0	2 (1.2; 5.3)	0
CNS, *n* (%; %^a^)	35 (20.9)	13 (7.8; 10.1)	22 (13.2; 57.9)	14 (8.4; 73.7)
Meningeal	9 (5.4)	0	9 (5.4; 23.7)	3 (1.8; 15.8)
Brain/spinal cord	22 (13.2)	13 (7.8; 10.1)^b^	9 (5.4; 23.7)	7 (4.2; 36.8)
Unknown^c^	4 (2.4)	0	4 (2.4; 10.5)	4 (2.4; 21.1)
GU, *n* (%; %^a^)	37 (22.2)	33 (19.8; 25.6)	4 (2.4; 10.5)	3 (1.8; 15.8)
Abnormal uterine bleeding	23 (13.8)	22 (13.2; 17.1)	1 (0.6; 2.6)	3 (1.8; 15.8)^d^
Hematuria	12 (7.2)	10 (6; 7.8)	2 (1.2; 5.3)	0
Hemospermia	1 (0.6)	1 (0.6; 0.8)	0	0
Vulvar hemorrhage	1 (0.6)	0	1 (0.6; 2.6)	0
Abdominal, *n* (%; %^a^)	21 (12.6)	13 (7.8; 10.1)	8 (4.8; 21.1)	2 (1.2; 10.5)
Upper GI tract	6 (3.6)	4 (2.4; 3.1)	2 (1.2; 5.3)	0
Lower GI tract	6 (3.6)	6 (3.6; 4.7)	0	0
Intra-abdominal	4 (2.4)	1 (0.6; 0.8)	3 (1.8; 7.9)	1 (0.6; 5.3)
Retroperitoneal	3 (1.8)	0	3 (1.8; 7.9)	1 (0.6; 5.3)
Unknown^c^	2 (1.2)	2 (1.2; 1.6)	0	0
Patients	80	49	31	15
% Total population^e^	40.6	24.9	15.7	7.6
% Bleeding event patients	100.0	61.3	38.8	18.8
% Major bleeding patients	—	—	—	48.4

Data are shown as number (%).

CNS, central nervous system; GI, gastrointestinal; GU, genitourinary.

a Prevalence within the group.

b All asymptomatic brain microhemorrhages found in brain imaging.

c Not detailed on clinical notes.

d All arising from CRNM-BE.

e *N* = 197.

### 3.2 Bleeding events: contributing factors

Table 4 provides a summary of the contributing factors for all bleeding events. Supplementary Tables S2 and S3 detail the clinical and demographic characteristics and surrounding circumstances for all major (events: *n* = 38; patients: *n* = 31) and nonmajor bleeding events (events: *n* = 129; patients: *n* = 67). On those tables, data are organized chronologically, by bleeding event per patient, and hence, the total number of affected patients differs from that in Table 3. Thrombocytopenia was present in 28.1% (47/167) of all bleeding events and occurred in 25% (20/80) of patients affected by any bleeding event (10.2% [20/197] of all patients). Thrombocytopenia was classified as mild, moderate, and severe in 29.8% (14/47), 34.0% (16/47), and 21.3% (10/47) of bleedings, respectively. Thrombocytopenia was present in 34.2% (13/38) of major bleeding events and in 26.4% (34/129) of nonmajor bleeding events. When present, thrombocytopenia was likely involved in 62.2% (23/37) of all hemorrhagic complications (major bleeding events: 72.7% [8/11]; nonmajor bleeding events: 57.7% [15/26]) occurring in 70.6% (12/17) of patients with thrombocytopenia (Table 4).

**Table 4:** Period of diagnosis and prevalence of contributing factors for bleeding events.

Variables	Total Bleeding events | patients	Nonmajor bleedings Bleeding events| patients	Major bleedings Bleeding events | patients
Total	167 | 80	129 | 49	38 | 31
Thrombocytopenia, *n* (%; %^a^)	47 (28.1) | 20 (25.0)	34 (26.4) | 11 (22.4)	13 (34.2) | 9 (29.0)
Likely involved in bleeding	23/37 (62.2) | 12/17 (70.6)	15/26 (57.7) | 6/9 (66.7)	8/11 (72.7) | 6/8 (75.0)
Mild (100-150 × 10^9^ platelets/L)	14 (8.4; 29.8) |5 (6.25; 25.0)	12 (9.3; 35.3) | 4 (8.2; 36.4)	2 (5.3; 15.4) | 1 (3.2; 11.1)
Likely involved in bleeding	5/10 (50.0) | 3/4 (75.0)	5/8 (62.5) | 3 (75.0)	— | —
Moderate (50-100 × 10^9^ platelets/L)	16 (9.6; 34.0) | 7 (8.75; 35.0)	9 (7.0; 26.5) | 2 (4.1; 18.2)	7 (18.4; 53.8) | 5 (16.1; 55.6)
Likely involved in bleeding	10 (62.5) | 4 (57.1)	5/7 (71.4) | 1 (50.0)	5 (62.5) | 3 (60.0)
Severe (<50 × 10^9^ platelets/L)	10 (6.0; 21.3) | 5 (6.25; 29.4)	8 (6.2; 23.5) | 3 (6.1; 27.3)	2 (5.3; 15.4) | 2 (6.5; 22.2)
Likely involved in bleeding	5 (50.0) | 4 (80.0)	3 (37.5) | 2 (66.7)	2 (100.0) | 2 (100.0)
Unknown^b^	7 (4.2; 14.9) | 3 (3.75; 15.0)	5 (3.9; 14.7) | 2 (18.2)	2 (5.3; 15.4) | 1 (11.1)
Likely involved in bleeding	3/5 (60.0) | 1/1 (100.0)	2/3 (66.7) | —	1 (50.0) | 1 (100.0)
Dual therapy, *n* (%; %^a^)	31/152 (20.4) | 16 (20.0)	22/116 (18.9) | 8 (16.3)	9/36 (25.0) | 8 (25.8)
Warfarin + low-dose aspirin	19 (12.5; 61.3) | 10 (12.5; 62.5)	13 (11.2; 59.1) | 4 (8.2; 50.0)	7 (19.4; 77.8) | 6 (19.4; 75.0)
Warfarin + clopidogrel	11 (7.2; 35.5) | 5 (6.25; 31.3)	8 (6.9; 36.4) | 3 (6.1; 37.5)	2 (5.6; 22.2) | 2 (6.5; 25.0)
LMWH + low-dose aspirin	1 (0.7; 3.2) | 1 (1.3; 6.3)	1 (0.9; 4.5) | 1 (2.0; 12.5)	0 | 0
Spontaneous, *n* (%; %^a^)			
Yes	151 (90.4) | 65 (81.3)	125 (96.9) | 46 (93.9)	26 (68.4) | 19 (61.3)
No	16 (9.6) | 15 (18.7)	4 (3.1) | 3 (6.1)	12 (31.6) | 12 (38.7)
Trauma	7 (4.2; 43.8) | 6 (7.5; 40.0)	2 (1.6; 50.0) | 1 (2.0; 33.3)	5 (13.2; 41.7) | 5 (16.1; 41.7)
Iatrogenic	9 (5.4; 56.3) | 9 (11.3; 60.0)	2 (1.6; 50.0) | 2 (4.1; 66.7)	7 (18.4; 58.3) | 7 (22.6; 58.3)
Surgery	4 (2.4; 44.4) | 4 (5.0; 44.4)	0 | 0	4 (10.5; 57.1) | 4 (12.9; 57.1)
Biopsy	2 (1.2; 22.2) | 2 (2.5; 22.2)	1 (0.8; 50.0) | 1 (2.0; 50.0)	1 (2.6; 14.3) | 1 (3.2; 14.3)
Other medical procedure^c^	3 (1.8; 33.3) | 3 (3.8; 33.3)	1 (0.8; 50.0) | 1 (2.0; 50.0)	2 (5.3; 28.6) | 2 (6.5; 28.6)
Diagnosis per decade, *n* (%)^d^			
1990-1999	7 (4.2) | 5 (6.3)	3 (2.3) | 1 (2.0)	4 (10.5) | 4 (12.9)
2000-2009	31 (18.6) | 20 (25.0)	20 (15.5) | 9 (18.4)	11 (28.9) | 11 (35.5)
2010-2019	110 (65.9) | 62 (77.5)	87 (67.4) |41 (83.7)	23 (60.5) | 21 (67.7)
Unknown^e^	19 (11.4) | 10 (12.5)	19 (14.7) | 10 (20.4)	0 | 0

Data are shown as number (%) and all data are shown when available. The denominators are provided if they differed from the overall numbers within the group.

BE, bleeding events; LMWH, low-molecular-weight heparin.

a Prevalence within the group.

b We were able to define the likelihood of contribution of unknown value thrombocytopenia to the bleeding event according to temporal concordance.

c Other medical procedure included pacemaker implantation (*n* = 1), ureteral stent implantation (*n* = 1), and pulmonary endarterectomy (*n* = 1).

d The number of affected patients within each decade may vary from the overall total as a patient may experience >1 bleeding event in different years (see Supplementary Tables S2 and S3 for further details).

e Not specified in medical notes.

Dual antithrombotic therapy was used in 20.4% (31/152) of all bleeding events, 61.3% (19/31) of them composed by warfarin and low-dose aspirin (LDA) and 35.5% (11/31) by warfarin and clopidogrel. In the whole group, only 5 patients (ID: 16, 23, 79, 184, and 211) had thrombocytopenia and were treated with combined antithrombotic therapy near the event (Supplementary Tables S2 and S3).

When applicable (ie, excluding all bleeding events occurring in patients receiving antiplatelet agents only, *n* = 11), the intensity of anticoagulation at the time of the bleed was only reported in 17.9% (28/156) of all bleedings (50.0% [18/36] of major bleeding events; 8.3% [10/120] of nonmajor bleeding events; calculated from Supplementary Tables S2 and S3). The INR value was known in 5.7% (9/156) of all bleeding events; it was >3 in 7 bleedings and >8 in 3 bleedings (ID: 65, 152, and 173). Only one of these patients (ID: 65) was also treated with combined antithrombotic therapy at the time of the event (Supplementary Table S3), and in only 1 patient (ID: 173) was thrombocytopenia a concomitant contributing factor to the bleeding (74 × 10^9^ platelets/L; Supplementary Table S2). Notably, 3 out of these 9 patients suffered from a major bleeding event affecting the CNS.

Most bleeds occurred spontaneously (90.4%). Notably, the frequency of nonspontaneous bleeds differed greatly between major (31.6% [12/38]) and nonmajor bleeding events (3.1% [49/129]). In 56.3% (9/16) of cases, they followed or were associated with medical procedures, mainly surgery (44.4% [4/9]), followed by trauma (43.8% [7/16]).

### 3.3 Damage acquisition

Damage occurred in 11.4% (19/167) of all bleeding events affecting 7.6% (15/197) of all patients. When considering only major bleeding events, we observed damage events in 50.0% (19/38) of cases (Table 3). The descriptive analysis of the damage events is shown in Table 5. The CNS contributed to 73.7% (14/19) of damage events occurring in 5.1% (10/197) of all patients. Most of the damage arose from major bleeding events that affected the brain, cerebellum, and/or spinal cord (events: *n* = 7; patients: *n* = 5), leading to permanent motor and/or sensory deficits. Notably, it was the cause of death in 1 patient (ID: 143; Table 5). After the CNS, 15.8% (3/19) of damage was related to GU bleedings, affecting 3 patients who experienced 3 CRNM bleedings. All these patients experienced severe abnormal uterine bleeding, indicating hysterectomy as the cause of damage in this domain. Bleeding events affecting the abdominal compartment led to 10.5% (2/19) of damage events affecting 1% of all patients (2/197). Both patients experienced an intra-abdominal hemorrhage leading to organ resection (Table 5). No damage event was observed in soft/connective tissue domain.

**Table 5:** Damage specification.

Affected organ/system		Damage specification	Damage events	Affected patients
Central nervous system	Brain/cerebellum/spinal cord	Seizures	1	5
		Motor deficits	4	
		Sensory deficits	2	
	Meningeal (subdural and/or subarachnoid)	Motor deficits	3	3
	Unknown	Seizures	1	3
		Motor deficit	1	
		Sensory deficits	1	
		Cause of death	1	
Gynecologic		Abnormal uterine bleeding leading to hysterectomy	3	3
Abdominal		Massive intra-abdominal hemorrhage leading to oophorectomy and salpingectomy	2	2
		Splenectomy during colonic surgery		

Patient ID 148 (Supplementary Table S2) suffered from 2 major bleedings affecting the central nervous system: 1 affecting the brain and other of unknown origin. Thus, the total number of affected patients presented here (*n* = 16) differs from those in Table 3 (*n* = 15).

## 4 Discussion

In this study, we have described the prevalence and severity of hemorrhagic complications according to the ISTH definitions [[13], [14], [15]] and detailed the damage acquisition related to bleeding events in a well-characterized group of patients with APS. We found that 40% of patients experienced at least 1 bleeding event during follow-up, and almost 8% were left with permanent disability that is not recognized in the current version of DIAPS.

The incidence of bleeding events, their severity, and consequences in thrombotic APS have been hampered not only by the paucity of published information but also by the heterogeneity of the definitions used. Thus, standardization of the definition of bleeding events, regardless of whether they are minor or major, is crucial to facilitate valid comparisons of the rates of bleeding between studies and to raise awareness of the need for improvement of APS treatment. The prevalence of bleeding events in warfarin-treated patients with nonvalvular atrial fibrillation is approximately 44% [16]. In the study by Dall’Ara et al. [5], 34% of patients experienced at least 1 bleeding, which were classified as mild hemorrhages in 26% of patients. The size and characteristics of our sample differ greatly from theirs, but we describe similar findings as 40.6% of all patients suffered from bleeding events and 24.9% were classified as nonmajor. The distribution of organ affection also follows similar patterns, with soft tissue hemorrhage/easy bruising, epistaxis, and menorrhagia being the most frequently reported manifestations. By definition, a minor bleeding event does not affect mortality [13]. Nevertheless, bleeding events secondary to antithrombotic agent are associated with health-related quality of life impairment [17], especially among patients treated with warfarin [16]. In our experience, its recognition and clear discussion with the patient can help identify and minimize additional risk factors associated with increased bleeding risk and help making informed treatment decisions both for the treating physician and the patient.

Eight percent of patients in the study by Dall’Ara et al. [5] suffered from what they classify as a severe hemorrhagic complication. According to the authors, subdural hematoma, severe menorrhagia, and esophageal varice bleeding were the consequences of those events [5]. In the prospective study by Serrano et al. [6], 9.4% of patients experienced 15 “notable” bleeding episodes. The CNS (*n* = 4, 26.7%), GI (*n* = 4, 26.7%), and GU (*n* = 2, 13.3%) systems were also the most frequently affected. In both cases, the criteria used to classify these hemorrhages were not specified. According to the ISTH definitions of major bleeding in nonsurgical patients [14], any symptomatic bleeding in a critical area or organ, such as intracranial or intraspinal, should be classified as major bleeding. Hence, at least some of their patients would still have been classified as having had a severe hemorrhagic complication had these definitions been used. In contrast, an upper gastrointestinal bleeding or abnormal uterine bleeding, regardless of the impact and apparent severity that its clinical presentation might have, is not to be classified as a major bleeding event unless it causes a fall in hemoglobin level of at least 2 g/dL, needs a blood transfusion, or is fatal. This information is lacking in those studies and would facilitate contextualization. Even though clinical judgment is hardly reproducible and can lead to bias, our results are in line with this pattern of organ affectation; the CNS was the most affected system, followed by GI and GU systems.

Compared with other studies [5,6], we do observe a greater prevalence of severe bleeding events in our study as almost 16% of our patients experienced at least 1 major bleeding event during the follow-up time. Apart from the aforementioned differences in the methodology on how data were collected and bleeding events were classified, 1 striking characteristic of the enrolled patients differs between our study and other APS studies [5,6]. Only 51% of patients in the study by Dall’Ara et al. [5] and ∼73% of patients in the study by Serrano et al. [6] were classified as having thrombotic APS. Moreover, in the latter, 79.5% of patients received anticoagulation and only 5.1% received anticoagulant and LDA [6]. This finding contrasts with our findings as we only included patients with thrombotic APS, and all of them had been exposed to antithrombotic therapy during follow-up (98.5% anticoagulation; ∼25% anticoagulation and LDA). Nevertheless, our incidence rate for major bleeding events (1.6 events per 100 person-years) falls within the range reported in those patients prescribed VKAs for atrial fibrillation (0.9-3.4 per 100 patients-years) [18]. Using the ISTH definitions, the incidence rate of major bleedings in warfarin-treated atrial fibrillation was 3.09 events per 100 patients-year in a large randomized clinical trial including 9081 patients in the warfarin group [19]. Older age is associated with increased bleeding risk during warfarin treatment [20], and this might explain the higher incidence of major bleeding in atrial fibrillation. The bleeding risk also increases with the intensity of treatment and/or concomitant use of several antithrombotic agents. Compared with patients receiving anticoagulation alone, the combination of anticoagulant with antiplatelet agents increases the risk of bleeding by approximately 2-fold [21]. In our study, 20.4% of all bleeding events occurred in patients receiving dual antithrombotic therapy at the time of bleeding. Anticoagulation intensity is also critical to consider as the bleeding risk increases if higher INR levels (>3) are used [22]. In this context, Ruiz-Irastorza et al. [23] described an incidence rate of 6 major bleeds per 100 person-year in their series of 66 patients with thrombotic APS treated with a target INR range of 3 to 4. Unfortunately, we could only ascertain the INR value near the bleed in a minority of patients, mainly related to major bleeding events.

Currently, there is no definition of damage arising from bleeding events in patients with thrombotic APS and the DIAPS does not recognize these events in their definitions [11]. While accepting the general approach of the DIAPS, recognizing damage as being a permanent decrease in organ function lasting for at least 6 months [11], we also consider that a bleeding event leading to total or partial organ resection should count as damage. Our analysis highlighted that a hysterectomy performed in cases of severe abnormal uterine bleeding not otherwise controlled with noninvasive procedures could also count as damage. This is particularly the case in females of child-bearing potential in whom antithrombotic therapy should not be withdrawn due to their thrombotic condition. This is the case of each of the 3 patients (cases 62, 66, and 69) who had this damage item counted in their GU system, having had hysterectomy at the age of 30, 36, and 34 years, respectively. Over the past several years, the DIAPS has been increasingly reported as an outcome of interest; the pattern of damage acquisition has been explored [9] and its correlation with health-related quality of life has been investigated [24,25]. Medina et al. [25] observed that the DIAPS neuropsychiatric domain was the most frequently affected in a group of 67 patients with primary APS followed for a mean time of 15 years. The authors describe that the damage accrual correlated inversely and significantly with health-related quality of life, mainly affecting the physical component domain [25]. Notably, in our study, most damage related to bleeding events affected the CNS. The brain, cerebellum, and spinal cord were the most affected (7 damage events) and, in most cases, led to new and/or exacerbation of motor deficits. As APS predominantly affects young individuals, prevention of damage, whether associated with thrombotic or bleeding events, should be a major focus for physicians managing these patients.

The thrombotic vs bleeding risk assessment remains a challenging issue. Thrombotic APS is a chronic and recurrent condition and damage may well increase over time despite appropriate treatment [4]. Thus, current recommendations advise long-term treatment with antithrombotic therapy for secondary prophylaxis after an initial thrombotic event [2]. In contrast, bleeding risk and its possible associated damage should raise questions concerning treatment modalities, their life-long maintenance, and the need to standardize bleeding risk assessments. When compared with VKA, the advantages of direct oral anticoagulants include reduced major and intracranial bleeding [26]. However, 2 meta-analyses of randomized controlled trials [27,28] in patients with APS showed a significantly higher risk of arterial thrombosis during treatment with DOACs than with warfarin (odds ratio: 5.17 [95% CI: 1.57-17.04] and 5.43 [95% CI: 1.87-15.75], respectively), though the risk of venous thromboembolism was not increased. Recently, the possible discontinuation of anticoagulant therapy in patients with primary APS in whom aPL become persistently negative has been discussed, but there are still not enough data to make a formal recommendation [29].

The strength of this study is the detailed description of all cumulative bleeding complications and, most importantly, the assessment of damage accrual associated with bleeding events in a relatively large real-world group of patients. The main limitations are related to the retrospective nature of the study and the long period of time during which we saw these patients. First, we cannot be absolutely certain that all the patients were recruited consecutively. This applies mainly for those patients with >20 years of follow-up and for whom only paper medical records existed (ie, those who died before any electronic system was implemented [in 2003]). We can provide reassurance that all the patients whose records were identified were thoroughly analyzed. Hence, our group of patients includes all patients under follow-up in the rheumatology and hematology clinics who met the inclusion and exclusion criteria (ie, before any knowledge about bleeding complications). Moreover, patients with APS, especially those with thrombotic phenotypes as those included here, require life-long follow-up in specialist centers, such as our own dedicated clinics. In this regard, we acknowledge that it is not our practice (past and current) to discharge these patients (ie, refer them back to the general practitioner) when they are stable. Thus, a selection bias is unlikely. However, we suspect that attrition bias may have affected the data as the cumulative incidence of any bleeding of >75% after 30 years using the Kaplan-Meier estimate is much higher than the crude proportion of patients who developed bleeding (41%). While believing that a long follow-up period is crucial to capture a real-world clinical events, it is worth noting that a minority of patients were followed for such an extended duration (follow-up time: ≥30 years, *n* = 6, 3.0%). As a result, the Kaplan-Meier curves probably provide an overestimated cumulative incidence, which may not necessarily reflect what would have been observed if all patients had been followed for the same length of time. Second, even though we undertook a thorough analysis of all medical records, the real circumstances of the bleeding events, mainly related to the treatment regimen, the intensity of the anticoagulation at the time of the event, and time in target INR range were not specified for all patients. Third, other independent risk factors for bleeding (eg, uncontrolled arterial hypertension and malignancy) were not specified. In this regard, we also acknowledge that the risk of bleeding may have changed over time due to therapeutic approaches, improved medical expertise, improved diagnostic testing, and management of bleeding. Thus, our results may not accurately reflect the current bleeding risk and bleeding-associated damage of patients with APS. Fourth, we could not ascertain whether all bleedings were recorded in medical records, especially in the older records that dated back to the 90s. Unless systematically asked about, minor hemorrhages (eg, easy bruising, gum bleeding, and epistaxis) could easily be missed in daily practice. Finally, being from a single center makes it hard to generalize our results. Future prospective studies should overcome these caveats.

## 5 Conclusion

Approximately 40% of patients with thrombotic APS experience at least 1 hemorrhagic event during their follow-up. According to the ISTH definitions, more than two-thirds of those are classified as major event and lead to permanent disability in >18% of patients affected by any bleeding event. The neurologic system was the most frequently affected by major hemorrhages, leading to damage accrual. As awareness of APS-related damage and its implications on quality of life and mortality are increasingly reported, further studies are needed in order to validate our findings and support the inclusion of damage caused by bleeding events in future revision of the DIAPS.
