# Supplementary material for: Bleeding events in thrombotic antiphospholipid syndrome: prevalence, severity, and associated damage accrual
Source: Res Pract Thromb Haemost. 2024 Jan 26;8(1):102327. doi: 10.1016/j.rpth.2024.102327 (PMC10879794; doi:10.1016/j.rpth.2024.102327)
Supplement: Supplementary Table [file mmc1.docx]

**Supplementary Tables**

**Supplementary Table 1.** Demographic and clinical characterization organized by follow-up time.

|  | **Total** | **<10 years** | **≥10 years** | **≥20 years** | **≥30 years** |
| --- | --- | --- | --- | --- | --- |
| **Patients**, n (%) | 197 (100.0) | 95 (48.2) | 102 (51.8) | 34 (17.3) | 6 (3.0) |
| **Female sex**, n (%) | 140 (71.1) | 62 (65.3) | 78 (76.5) | 31 (91.2) | 5 (83.3) |
| **Type of APS**, n (%) |  |  |  |  |  |
| Primary | 130 (65.9) | 63 (66.3) | 67 (65.7) | 23 (67.7) | 5 (83.3) |
| Secondary | 67 (34.0) | 32 (33.7) | 35 (34.3) | 11 (32.4) | 1 (16.7) |
| Systemic Lupus Erythematosus | 57 (28.9) | 26 (27.4) | 31 (30.4) | 10 (29.4) | 1 (16.7) |
| Other * | 10 (5.1) | 6 (6.3) | 4 (3.9) | 1 (2.9) | 0 |
| **Age at APS onset** (y) | 40 (28 - 51) | 43 (34 - 53) | 36 (26 - 49) | 36 (27 - 42) | 20 (17 - 27) |
| **Type of first event**, n (%) |  |  |  |  |  |
| Venous | 116 (58.8) | 59 (62.1) | 57 (55.9) | 20 (58.8) | 4 (66.7) |
| Arterial | 87 (44.2) | 39 (41.1) | 48 (47.1) | 16 (47.1) | 3 (50.0) |
| Microvascular ^†^ | 5 (2.5) | 2 (1.0) | 3 (2.9) | 0 | 0 |
| **Treatment regimen (ever)**, n (%) |  |  |  |  |  |
| Anticoagulation and/or antiplatelet therapy | 197 (100.0) | 95 (100.0) | 102 (100.0) | 34 (100.0) | 6 (100.0) |
| Anticoagulation | 194 (98.5) | 95 (100.0) | 99 (97.1) | 32 (94.1) | 5 (83.3) |
| Antiplatelet therapy | 52 (26.4) | 15 (15.8) | 37 (36.3) | 15 (44.1) | 3 (50.0) |
| Anticoagulation and antiplatelet therapy | 49 (24.9) | 15 (15.8) | 34 (33.3) | 13 (38.2) | 2 (33.3) |
| **Patients affected by bleeding events (ever)**, n (%) |  |  |  |  |  |
| Any bleeding event | 80 (40.6) | 29 (30.5) | 51 (50.0) | 18 (52.9) | 3 (50.0) |
| Major bleeding event | 31 (15.7) | 8 (8.4) | 23 (22.5) | 9 (26.5) | 2 (33.3) |
| **Final DIAPS** |  |  |  |  |  |
| Median (IQR) | 1 (0 - 2) | 1 (0 - 2) | 1 (1 - 3) | 1 (1 - 3) | 4.5 (3 - 8) |
| Mean ± SD | 1.6 ± 1.7 | 1.2 ± 1.3 | 1.9 ± 1.9 | 2.0 ± 2.1 | 4.8 ± 2.8 |
| **Death** | 23 (11.7) | 9 (9.5) | 14 (13.7) | 4 (11.8) | 0 |

Data are shown as number (%) for categorical variables, and median (interquartile range, [IQR]) for continuous variables. DIAPS, Damage Index for APS; y, years.

* Other include the following: Rheumatoid arthritis (n=3, 1.5%), undifferentiated autoimmune rheumatic disease (n=5, 2.5%), psoriatic arthritis (n=1, 0.5%), and rhupus (n=1, 0.5%). † Biopsy proven.

Supplementary Table 2. Characterization of major bleeding events.

| Demographic and clinical characteristics | | | | | | Thrombocytopenia | | Treatment regimen | | | Treatment intensity | | Circumstances | | Bleeding event | | |
| --- | --- | --- | --- | --- | --- | --- | --- | --- | --- | --- | --- | --- | --- | --- | --- | --- | --- |
| ID | **Sex** | **Type \| Condition** | **Age (y) \| Onset (y)** | **Follow-up (y)** | **Thrombosis recurrence *** | **Severity †, ‡ \| PTL count §** | **Likely involved in bleeding** | **Near bleeding** | **Ever** | **Last ¶** | **Last **** | **Near bleeding** | **Spontaneous** | **Comorbid conditions** | **Manifestations (y)** | **Affected system** | **Damage** |
| 16 | F | PAPS \| - | 12 \| 1987 | 32 | Arterial | Severe ITP with splenectomy (1998) \| - | Yes | - | W, AAS, Clopi | W, Clopi | 3.5 - 4.5 | - | Yes | - | Subarachnoid haemorrhage (1998) | CNS | No |
| 21 | F | PAPS \| - | 40 \| 1999 | 20 | No | No \| 362 | No | W | W | W | 2 - 3 | - | No | Vulvar biopsy | Haemorrhagic shock (2011) | GU | No |
| 46 | F | PAPS \| - | 54 \| 2001 | 18 | No | No \| 223 | No | LMWH | W | W | 2.5 - 3.5 | Standard | No | Post-operative | Bleeding while on LMWH bridging needing blood transfusion (2010) | Soft tissue | No |
| 51 | M | PAPS \| - | 33 \| 1992 | 27 | Arterial | No \| 323 | No | W, AAS | W, AAS | W, AAS | 4 - 4.5 | - | Yes | - | Muscular hematoma (2019) | Soft tissue | No |
| 58 | F | PAPS \| - | 20 \| 2014 | 5 | Venous | No \| 416 | No | RVX | W, RVX | RVX | 2 - 3 | 20mg | No | Ureteral stent implantation | Haematuria needing blood transfusion (2016) | GU | No |
| 65 | M | PAPS \| - | 44 \| 2011 | 8 | No | Mod. \| 160 | No | W | W | W | 2 - 3 | >10 | Yes | Oesophagitis | Upper GI bleeding needing transfusion and ICU admission (2018) | Abdominal | No |
| 69 | F | PAPS \| - | 43 \| 2009 | 10 | Venous | No \| 253 | No | LMHH, AAS | W, LMWH, AAS | W, AAS | 3 - 4 | Standard | No | Minor trauma | Hemarthrosis (2010) | Soft tissue | No |
| 82 | M | PAPS \| - | 51 \| 1991 | 28 | No | No \| - | No | W | W | W | 2 - 3 | - | Yes | - | Subdural hematoma (2001) | CNS | No |
| 96 | F | PAPS \| - | 42 \| 2002 | 12 | No | No \| 279 | No | W, AAS | W, AAS | W, AAS | 2 - 3 | - | Yes | - | Subdural hematoma (2007) | CNS | No |
| 99 | F | PAPS \| - | 26 \| 2002 | 17 | Venous | Mod. \| 60 | Yes | LMWH | W, LMWH | LMWH | 2 -3 | Standard | Yes | - | Subdural and brain hematoma (2016) | CNS | Yes (2x) |
| 108 | F | PAPS \| - | 29 \| 2000 | 19 | No | No \| 279 | No | LMWH | LMWH | LMWH | Standard | Standard | Yes | - | Haematuria needing blood transfusion (2019) | GU | No |
| 112 | M | PAPS \| - | 43 \| 2012 | 6 | No | No \| 219 | No | W | W | W | 2.5 - 3.5 | 3 - 4 | No | Trauma | Epidural hematoma (2015) | CNS | No |
| 128 | F | PAPS \| - | 31 \| 2006 | 6 | No | No \| 323 | No | W | W | W | 2 - 3 | 1.5 | Yes | - | Cerebellar hematoma (2007) | CNS | Yes |
| 143 | F | SAPS \| SLE | 32 \| 2001 | 13 | Arterial | No \| - | No | W, Clopi | W, Clopi | W, Clopi | 3 - 4 | - | Yes | - | CNS haemorrhage (***cause of death***) (2014) | CNS | Yes |
| 148 | M | SAPS \| SLE | 53 \| 1991 | 15 | No | ITP unknown severity (since 2000) \| - | No | W | W, AAS | AAS | 3 - 4 | - | Yes | - | Intraventricular haemorrhage requiring craniectomy (1997) | CNS | Yes |
| 148 | M | SAPS \| SLE | 53 \| 1991 | 15 | No | ITP unknown severity (since 2000) \| - | Yes | AAS | W, AAS | AAS | 3 - 4 | N/A | No | Minor trauma | CNS hematoma (2003) | CNS | Yes |
| 151 | F | SAPS \| SLE | 59 \| 2007 | 11 | No | Severe \| 33 | Yes | AAS | LMWH, AAS | AAS | N/A | N/A | Yes | - | Subdural hematoma (2017) | CNS | No |
| 152 | F | SAPS \| SLE | 22 \| 1990 | 29 | No | No \| - | No | W | W | W | 2 - 3 | 8 - 9 | Yes | - | CNS haemorrhage (unknown origin) (1990) | CNS | No |
| 152 | F | SAPS \| SLE | 22 \| 1990 | 29 | No | No \| 226 | No | W | W | W | 2 - 3 | - | Yes | Angiolipoma | Retroperitoneal hematoma (2014) | Abdominal | No |
| 153 | F | SAPS \| SLE | 17 \| 1980 | 39 | Arterial and venous | No \| - | No | W | W | W | - | - | Yes | - | Retroperitoneal haemorrhage (2000) | Abdominal | No |
| 161 | F | SAPS \| SLE | 45 \| 1997 | 21 | No | No \| - | No | - | W, AAS | W, AAS | - | - | No | Post-pulmonary endarterectomy | CNS haemorrhage (unknown origin) (2001) | CNS | Yes (2x) |
| 172 | F | SAPS \| SLE | 38 \| 2014 | 5 | No | No \| 343 | No | W | W, RVX | RVX | 2 - 3 | 2.4 | No | Surgery | Retroperitoneal hematoma and splenectomy after colonic surgery (2016) | Abdominal | Yes |
| 173 | F | SAPS \| SLE | 21 \| 2004 | 15 | Venous | Mod. \| 74 | Yes | W | W | W | 3 - 4 | 8 | Yes | - | Subdural hematoma (2018) | CNS | Yes |
| 175 | F | SAPS \| SLE | 46 \| 2013 | 6 | No | No \| 263 | No | LMWH | W, LMWH | W | 3 - 4 | Standard | No | Surgery | Life-threatening post-operative intra-abdominal bleeding requiring emergent laparotomy (2013) | Abdominal | No |
| 182 | F | SAPS \| SLE | 25 \| 2001 | 18 | Arterial, venous, and microvascular | Mod. \| 64 | Yes | LMWH | W, LMHW | LMWH | 2 - 3 | Standard | Yes | - | Subdural hematoma and subarachnoid haemorrhage (2016) | CNS | Yes |
| 182 | F | SAPS \| SLE | 25 \| 2001 | 18 | Arterial, venous, and microvascular | Mod. \| 66 | Yes | LMWH | W, LMHW | LMWH | 2 - 3 | Standard | Yes | - | Subdural hematoma (2017) | CNS | No |
| 182 | F | SAPS \| SLE | 25 \| 2001 | 18 | Arterial, venous, and microvascular | Mod. \| 54 | Yes | LMWH | W, LMHW | LMWH | 2 - 3 | Standard | Yes | - | Subarachnoid haemorrhage and cerebellar hematoma (2018) | CNS | Yes |
| 183 | F | SAPS \| SLE | 39 \| 2004 | 15 | Arterial | No \| - | No | W | W | W | 2 - 3 | - | Yes | - | Brain hematoma (2006) | CNS | No |
| 184 | F | SAPS \| SLE | 26 \| 2012 | 7 | No | Mod. \| 194 | No | W, AAS | W, AAS | W, AAS | 3 - 4 | - | Yes | - | AUB requiring blood transfusion (2012) | GU | No |
| 186 | F | SAPS \| SLE | 30 \| 1994 | 25 | Venous | No \| 297 | No | FPNX | W, FPNX | FPNX | Standard | Standard | Yes | - | CNS haemorrhage (unknown origin) (2013) | CNS | Yes |
| 189 | F | SAPS \| SLE | 21 \| 2005 | 14 | Arterial | No \| - | No | W | W, AAS | W, AAS | 3 - 4 | - | Yes | Ovarian cyst rupture | Intra-abdominal bleeding requiring blood transfusion, oophorectomy, and salpingectomy (2009) | Abdominal | Yes |
| 191 | F | SAPS \| SLE | 16 \| 2004 | 15 | No | No \| - | No | W, AAS | W, AAS | W, AAS | 2 - 3 | - | Yes | - | Cerebellar hematoma (2004) | CNS | Yes |
| 191 | F | SAPS \| SLE | 16 \| 2004 | 15 | No | No \| - | No | W, AAS | W, AAS | W, AAS | 2 - 3 | - | No | Trauma | Hemarthrosis after trauma (2015) | Soft tissue | No |
| 196 | M | PAPS \| - | 71 \| 2011 | 1 | No | No \| 207 | No | LMWH | LMWH | LMWH | Standard | Standard | Yes | - | Upper GI bleeding needing blood transfusion (2012) | Abdominal | No |
| 204 | F | SAPS \| SLE | 42 \| 1997 | 22 | No | No \| - | No | W | W, Clopi | W, Clopi | 2 - 3 | - | Yes | - | Brain hematoma (1998) | CNS | No |
| 204 | F | SAPS \| SLE | 42 \| 1997 | 22 | No | No \| - | No | W, Clopi | W, Clopi | W, Clopi | 2 - 3 | - | No | Surgery | Intra-abdominal haemorrhage (2008) | Abdominal | No |
| 211 | M | SAPS \| UCTD | 53 \| 2000 | 19 | No | Mild \| - | - | W, AAS | W, LMWH, AAS | LMWH | 2 - 3 | - | No | Trauma | Subdural hematoma (2009) | CNS | No |
| 211 | M | SAPS \| UCTD | 53 \| 2000 | 19 | No | Mild \| - | - | LMWH | W, LMWH, AAS | LMWH | Prophylactic | Prophylactic | Yes | - | Brain and spinal cord hematoma (2010) | CNS | Yes |

Data are shown when available. Each row represents a distinct major-BE (n=**38**) affecting **31** thrombotic APS patients regardless of whether they were also affected by CRNM and/or minor-BE (**Supplementary Table 3**).

* Recurrent thrombotic events during antithrombotic therapy, regardless of the treatment modality and/or the intensity of anticoagulation; † Thrombocytopenia severity is stated as the lowest value ever present; ‡ Mild: 100x10^9^/L to 150x10^9^/L, Mod.: 50x10^9^/L to 100x10^9^/L, Severe: <50x10^9^/L; § Near bleeding event. Absolute platelet counts are shown whenever thrombocytopenia existed; ¶ Last treatment modality; ** Latest INR target value/aimed anticoagulation intensity. AAS, acetylsalicylic acid; AUB, abnormal uterine bleeding; Clopi, clopidogrel; CNS, central nervous system; CRNM, clinically relevant non-major-BE; F, female; FPNX, fondaparinux; GI, gastrointestinal; GU, genitourinary; ICU, intensive care unit; INR, international normalized ratio; ITP, immune thrombocytopenia purpura; LMWH, low molecular weight heparin; M, male; N/A, not applicable; PAPS, primary antiphospholipid syndrome; PTL, platelets; RVX, rivaroxaban; RA, rheumatoid arthritis; SAPS, secondary antiphospholipid syndrome; SLE, systemic lupus erythematosus; UCTD, undifferentiated connective tissue disease; W, warfarin.

Supplementary Table 3. Characterization of non-major bleeding events.

| Demographic and clinical characteristics | | | | | | Thrombocytopenia | | Treatment regimen | | | Treatment intensity | | Circumstances | | Bleeding event | | | |
| --- | --- | --- | --- | --- | --- | --- | --- | --- | --- | --- | --- | --- | --- | --- | --- | --- | --- | --- |
| ID | **Sex** | **Type \| Condition** | **Age (y) \| Onset (y)** | **Follow-up (y)** | **Thrombosis recurrence *** | **Severity †, ‡ \| PTL count §** | **Likely involved in bleeding** | **Near bleeding** | **Ever** | **Last ¶** | **Last **** | **Near bleeding** | **Spontaneous** | **Comorbid conditions** | **Manifestations (y)** | **Classification** | **Affected system** | **Damage** |
| 4 | M | PAPS \| - | 65 \| 2009 | 7 | Arterial | Mild \| 115 | Yes | LMWH | LMWH, AAS | LMWH, AAS | Standard | Standard | No | Colonic polyp excision | Lower GI bleeding with iron deficient anaemia (2010) | CRNM | Abdominal | No |
| 7 | M | PAPS \| - | 41 \| 2006 | 9 | No | No | No | W | W | W | 3 - 4 | - | Yes | - | Haematuria (2014) | Minor | GU | No |
| 9 | F | PAPS \| - | 29 \| 1999 | 20 | Venous | No | No | W | W | W | 3.5 - 4.5 | - | Yes | Endometriosis | AUB with chronic iron deficient anaemia (2007) | CRNM | GU | No |
| 10 | M | PAPS \| - | 36 \| 2001 | 18 | No | No | No | W | W | W | 2 - 3 | - | Yes | - | Hematospermia (2004) | Minor | GU | No |
| 10 | M | PAPS \| - | 36 \| 2001 | 18 | No | No | No | W | W | W | 2 - 3 | - | Yes | - | Gum bleeding (2014) | Minor | Mucocutaneous | No |
| 10 | M | PAPS \| - | 36 \| 2001 | 18 | No | No | No | W | W | W | 2 - 3 | - | Yes | - | Lower bowel bleeding (2016) | CRNM | Abdominal | No |
| 15 | F | PAPS \| - | 54 \| 2001 | 18 | Arterial | No | No | W, AAS | W, AAS | W, AAS | 2.5 - 3.5 | 6.8 | Yes | - | Severe bilateral epistaxis (2011) | CRNM | Mucocutaneous | No |
| 15 | F | PAPS \| - | 54 \| 2001 | 18 | Arterial | No | No | W, AAS | W, AAS | W, AAS | 2.5 - 3.5 | - | No | Pacemaker implantation | Muscular hematoma (2011) | CRNM | Soft tissue | No |
| 15 | F | PAPS \| - | 54 \| 2001 | 18 | Arterial | No | No | - | W, AAS | W, AAS | 2.5 - 3.5 | - | Yes | - | Haematuria | Minor | GU | No |
| 16 | F | PAPS \| - | 12 \| 1987 | 32 | Arterial | Severe ITP with splenectomy (1998) \| - | No | W, Clopi | W, AAS, Clopi | W, Clopi | 3.5 - 4.5 | - | Yes | - | Gum bleeding (2015) | Minor | Mucocutaneous | No |
| 16 | F | PAPS \| - | 12 \| 1987 | 32 | Arterial | Severe ITP with splenectomy (1998) \| - | No | W, Clopi | W, AAS, Clopi | W, Clopi | 3.5 - 4.5 | - | Yes | - | Epistaxis (2015) | Minor | Mucocutaneous | No |
| 18 | F | PAPS \| - | 37 \| 1999 | 20 | Arterial | Mod. \|75 (since 2016) | No | W | W, AAS | W, AAS | 2 - 3 | - | Yes | - | Haemoptysis (2003) | CRNM | Mucocutaneous | No |
| 19 | M | PAPS \| - | 36 \| 2006 | 14 | No | No | No | RVX | W, RVX, Clopi | RVX, Clopi | 2 - 3 | 20mg | Yes | Erosive duodenitis | Upper bowel bleeding with iron deficient anaemia (2016) | CRNM | Abdominal | No |
| 23 | M | PAPS \| - | 33 \| 1976 | 43 | Venous | Mild \| 105-125 | Yes | W | W, AAS | W, AAS | 3.5 - 4.5 | - | Yes | - | Easy bruising (2009) | Minor | Mucocutaneous | No |
| 23 | M | PAPS \| - | 33 \| 1976 | 43 | Venous | Mild \|105-125 | Yes | W | W, AAS | W, AAS | 3.5 - 4.5 | - | Yes | - | Gum bleeding (2009) | Minor | Mucocutaneous | No |
| 23 | M | PAPS \| - | 33 \| 1976 | 43 | Venous | Mild \|105-125 | Yes | W, ASS | W, AAS | W, AAS | 3.5 - 4.5 | - | Yes | - | Haemoptysis (2011) | CRNM | Mucocutaneous | No |
| 29 | F | PAPS \| - | 36 \| 2003 | 16 | Arterial | No | No | - | W, AAS | W, AAS | 3 - 4 | 5 | Yes | - | Muscular hematoma | CRNM | Soft tissue | No |
| 46 | F | PAPS \| - | 54 \| 2001 | 18 | No | No | No | W | W | W | 2.5 - 3.5 | - | Yes | - | CNS microhaemorrhages (2018) | Minor | CNS | No |
| 51 | M | PAPS \| - | 33 \| 1992 | 27 | Arterial | No | No | W, AAS | W, AAS | W, AAS | 4 - 4.5 | - | Yes | - | GI bleeding with iron deficient anaemia (1998) | CRNM | Abdominal | No |
| 51 | M | PAPS \| - | 33 \| 1992 | 27 | Arterial | No | No | - | W, AAS | W, AAS | 4 - 4.5 | - | Yes | - | Soft tissue hematoma | CRNM | Soft tissue | No |
| 51 | M | PAPS \| - | 33 \| 1992 | 27 | Arterial | No | No | - | W, AAS | W, AAS | 4 - 4.5 | - | Yes | - | Easy bruising | Minor | Mucocutaneous | No |
| 51 | M | PAPS \| - | 33 \| 1992 | 27 | Arterial | No | No | - | W, AAS | W, AAS | 4 - 4.5 | - | Yes | - | Gum bleeding | Minor | Mucocutaneous | No |
| 51 | M | PAPS \| - | 33 \| 1992 | 27 | Arterial | No | No | - | W, AAS | W, AAS | 4 - 4.5 | - | Yes | - | Haematuria | Minor | GU | No |
| 54 | F | PAPS \| - | 29 \| 2003 | 16 | No | No | No | RVX | W, RVX | RVX | 2 - 3 | - | Yes | - | AUB (2014) | CRNM | GU | No |
| 54 | F | PAPS \| - | 29 \| 2003 | 16 | No | No | No | - | W, RVX | RVX | 2 - 3 | - | Yes | - | Easy bruising | Minor | Mucocutaneous | No |
| 59 | F | PAPS \| - | 42 \| 2017 | 2 | Arterial | No | No | W | W | W | 3 - 4 | - | Yes | - | AUB (2019) | CRNM | GU | No |
| 62 | F | PAPS \| - | 40 \| 2000 | 19 | Arterial | Severe (lowest PTL value: 15) \|40-80 | Yes | W | W | W | 3 | - | Yes | - | AUB leading to hysterectomy (2009) | CRNM | GU | ***Yes*** |
| 65 | M | PAPS \| - | 44 \| 2011 | 8 | No | Mod. \| - | - | W | W | W | 2 - 3 | 5 | Yes | - | Haematuria (2015) | CRNM | GU | No |
| 66 | F | PAPS \| - | 36 \| 2016 | 3 | No | No | No | W, Clopi | W, Clopi | W, Clopi | 2 - 3 | - | Yes | - | Gum bleeding (2016) | CRNM | Mucocutaneous | No |
| 66 | F | PAPS \| - | 36 \| 2016 | 3 | No | No | No | W, Clopi | W, Clopi | W, Clopi | 2 - 3 | - | Yes | - | Epistaxis (2017) | CRNM | Mucocutaneous | No |
| 66 | F | PAPS \| - | 36 \| 2016 | 3 | No | No | No | W, Clopi | W, Clopi | W, Clopi | 2 - 3 | - | Yes | Uterine fibroid | AUB leading to hysterectomy (2019) | CRNM | GU | ***Yes*** |
| 67 | F | PAPS \| - | 37 \| 2011 | 8 | No | No | No | W | W | W | 3 - 4 | - | Yes | - | AUB (2018) | CRNM | GU | No |
| 69 | F | PAPS \| - | 43 \| 2009 | 10 | Venous | No | No | W | W, LMWH, AAS | W, AAS | 3 - 4 | - | Yes | Endometriosis; uterine fibroid | AUB leading to hysterectomy (2010) | CRNM | GU | ***Yes*** |
| 69 | F | PAPS \| - | 43 \| 2009 | 10 | Venous | No | No | W, AAS | W, LMWH, AAS | W, AAS | 3 - 4 | - | No | Minor trauma | Right foot hematoma (2017) | CRNM | Soft tissue | No |
| 69 | F | PAPS \| - | 43 \| 2009 | 10 | Venous | No | No | - | W, LMWH, AAS | W, AAS | 3 - 4 | - | Yes | - | Easy bruising | Minor | Mucocutaneous | No |
| 70 | M | PAPS \| - | 60 \| 2006 | 13 | No | No | No | W | W | W | 2 - 3 | - | Yes | - | Epistaxis needing one transfusion (2018) | CRNM | Mucocutaneous | No |
| 72 | F | PAPS \| - | 25 \| 2013 | 6 | No | No | No | RVX | RVX, LMWH | RVX | - | 20mg | Yes | - | Easy bruising (2014) | Minor | Mucocutaneous | No |
| 79 | F | PAPS \| - | 29 \| 2006 | 13 | No | Mild \|- | - | W, AAS | W, AAS | W, AAS | 2.5 - 3.5 | - | Yes | - | Epistaxis (2013) | Minor | Mucocutaneous | No |
| 79 | F | PAPS \| - | 29 \| 2006 | 13 | No | Mild \| 220 | No | W, AAS | W, AAS | W, AAS | 2.5 - 3.5 | - | No | Minor trauma | Muscular hematoma (2017) | CRNM | Soft tissue | No |
| 79 | F | PAPS \| - | 29 \| 2006 | 13 | No | Mild \| - | - | W, AAS | W, AAS | W, AAS | 2.5 - 3.5 | - | Yes | - | Easy bruising | Minor | Mucocutaneous | No |
| 80 | F | PAPS \| - | 30 \| 2012 | 7 | Arterial and venous | No | No | LMWH | W, LMHW, FPNX, AAS | FPNX, AAS | 3 - 4 | Standard | Yes | - | AUB (2014) | CRNM | GU | No |
| 80 | F | PAPS \| - | 30 \| 2012 | 7 | Arterial and venous | No | No | W | W, LMHW, FPNX, AAS | FPNX, AAS | 3 - 4 | - | Yes | - | Abdominal wall hematoma (2015) | CRNM | Soft tissue | No |
| 80 | F | PAPS \| - | 30 \| 2012 | 7 | Arterial and venous | No | No | - | W, LMHW, FPNX, AAS | FPNX, AAS | 3 - 4 | - | Yes | - | Easy bruising | Minor | Mucocutaneous | No |
| 82 | M | PAPS \| - | 51 \| 1991 | 28 | No | No | No | W | W | W | 2 - 3 | - | Yes | Bronchiectasis | Haemoptysis (2010) | CRNM | Mucocutaneous | No |
| 82 | M | PAPS \| - | 51 \| 1991 | 28 | No | No | No | W | W | W | 2 - 3 | - | Yes | Bronchiectasis | Haemoptysis (2013) | CRNM | Mucocutaneous | No |
| 82 | M | PAPS \| - | 51 \| 1991 | 28 | No | No | No | W | W | W | 2 - 3 | - | Yes | Bronchiectasis | Haemoptysis (2017) | CRNM | Mucocutaneous | No |
| 83 | F | PAPS \| - | 25 \| 2000 | 19 | No | No | No | W | W | W | 3 - 4 | - | Yes | - | AUB with chronic iron deficient anaemia (2016) | CRNM | GU | No |
| 83 | F | PAPS \| - | 25 \| 2000 | 19 | No | No | No | W | W | W | 3 - 4 | - | Yes | - | Epistaxis (2017) | Minor | Mucocutaneous; | No |
| 88 | M | PAPS \| - | 52 \| 2011 | 8 | No | No | No | W | W | W | 2 - 3 | - | Yes | Colonic polyp | Lower GI bleeding (2012) | CRNM | Abdominal | No |
| 88 | M | PAPS \| - | 52 \| 2011 | 8 | No | No | No | W | W | W | 2 - 3 | - | Yes | During cough | Muscular hematoma (2018) | CRNM | Soft tissue | No |
| 93 | F | PAPS \| - | 54 \| 2006 | 13 | Arterial | No | No | W, Clopi | W, Clopi | W, Clopi | 3 - 4 | - | Yes | - | Skin hematoma (2010) | Minor | Soft tissue | No |
| 93 | F | PAPS \| - | 54 \| 2006 | 13 | Arterial | No | No | W, Clopi | W, Clopi | W, Clopi | 3 - 4 | - | Yes | - | Easy bruising | Minor | Mucocutaneous | No |
| 94 | F | PAPS \| - | 28 \| 2009 | 10 | No | No | No | RVX | W, RVX | RVX | 2 - 3 | - | Yes | Uterine fibroid | AUB with chronic iron deficient anaemia (2017) | CRNM | GU | No |
| 96 | F | PAPS \| - | 42 \| 2002 | 12 | No | No | No | W | W, AAS | W, AAS | 2 - 3 | - | Yes | - | AUB (2009) | Minor | GU | No |
| 98 | M | PAPS \| - | 56 \| 2007 | 12 | No | No | No | W | W | W | 2 - 3 | - | Yes | Urolithiasis | Haematuria (2014) | CRNM | GU | No |
| 99 | F | PAPS \| - | 26 \| 2002 | 17 | Venous | Mod \| - | Yes | W | W, LMWH | LMWH | 2 - 3 | - | Yes | Stomach telangiectasias | Upper GI bleeding (2015) | CRNM | Abdominal | No |
| 102 | M | PAPS \| - | 54 \| 2002 | 15 | Arterial | No | No | W | W | W | 3 - 4 | - | Yes | - | Epistaxis (2008) | Minor | Mucocutaneous | No |
| 105 | F | PAPS \| - | 38 \| 1997 | 23 | No | No | No | W | W | W | 3 - 4 | - | Yes | - | CNS microhaemorrhages (2016) | Minor | CNS | No |
| 107 | F | PAPS \| - | 45 \| 1992 | 27 | No | No | No | W | W, AAS | W, AAS | 2 - 3 | - | Yes | - | Easy bruises (2014) | Minor | Mucocutaneous | No |
| 107 | F | PAPS \| - | 45 \| 1992 | 27 | No | No | No | W | W, AAS | W, AAS | 2 - 3 | - | Yes | - | CNS microhaemorrhages (2016) | Minor | CNS | No |
| 107 | F | PAPS \| - | 45 \| 1992 | 27 | No | No | No | W | W, AAS | W, AAS | 2 - 3 | - | Yes | - | Haematuria (2017) | Minor | GU | No |
| 108 | F | PAPS \| - | 29 \| 2000 | 19 | No | No | No | LMWH | LMWH | LMWH | Standard | Standard | Yes | - | Abdominal wall hematoma (2014) | CRNM | Soft tissue | No |
| 113 | F | PAPS \| - | 23 \| 2011 | 6 | No | No | No | W | W | W | 3 - 4 | - | Yes | - | Haemorrhagic transformation of old stroke without symptoms (seen in MRI) (2014) | Minor | CNS | No |
| 115 | M | PAPS \| - | 73 \| 2012 | 7 | Arterial | Mild \| - | - | W | W | W | 2.5 - 3.5 | - | Yes | Benign prostate hyperplasia | Haematuria (2012) | Minor | GU | No |
| 115 | M | PAPS \| - | 73 \| 2012 | 7 | Arterial | Mild \| 110 | Yes | W | W | W | 2.5 - 3.5 | - | Yes | Benign prostate hyperplasia | Haematuria (2016) | Minor | GU | No |
| 124 | F | PAPS \| - | 19 \| 1991 | 28 | No | No | No | W | W | W | 3 - 4 | - | Yes | Uterine polyps | AUB (2012) | CRNM | GU | No |
| 124 | F | PAPS \| - | 19 \| 1991 | 28 | No | No | No | W | W | W | 3 - 4 | - | Yes | - | Haematuria (2013) | Minor | GU | No |
| 124 | F | PAPS \| - | 19 \| 1991 | 28 | No | No | No | W | W | W | 3 - 4 | - | Yes | - | Lower GI bleeding (2018) | CRNM | Abdominal | No |
| 134 | F | PAPS \| - | 27 \| 1994 | 25 | No | No | No | AAS | AAS | AAS | N/A | N/A | Yes | Uterine fibroid | AUB (2012) | Minor | GU | No |
| 135 | F | PAPS \| - | 35 \| 1997 | 17 | No | No | No | W | W | W | 3 - 4 | - | Yes | - | Easy bruising (2010) | CRNM | Mucocutaneous | No |
| 135 | F | PAPS \| - | 35 \| 1997 | 17 | No | No | No | W | W | W | 3 - 4 | - | Yes | Endometriosis | AUB | Minor | GU | No |
| 137 | F | PAPS \| - | 63 \| 2000 | 19 | No | No | No | W | W | W | 2 - 3 | - | Yes | - | Epistaxis (2014) | Minor | Mucocutaneous | No |
| 138 | F | PAPS \| - | 69 \| 2016 | 3 | Arterial | No | No | W | W | W | 3 - 4 | - | Yes | - | Epistaxis | Minor | Mucocutaneous | No |
| 145 | F | SAPS \| SLE | 49 \| 1998 | 19 | No | No | No | W | W | W | 2 - 3 | - | Yes | Bronchiectasis | Haemoptysis (2005) | CRNM | Mucocutaneous | No |
| 145 | F | SAPS \| SLE | 49 \| 1998 | 19 | No | No | No | W | W | W | 2 - 3 | - | Yes | Bronchiectasis | Haemoptysis (2006) | CRNM | Mucocutaneous | No |
| 145 | F | SAPS \| SLE | 49 \| 1998 | 19 | No | No | No | W | W | W | 2 - 3 | - | Yes | Bronchiectasis | Haemoptysis (2007) | CRNM | Mucocutaneous | No |
| 145 | F | SAPS \| SLE | 49 \| 1998 | 19 | No | No | No | W | W | W | 2 - 3 | - | Yes | Bronchiectasis | Haemoptysis (2008) | CRNM | Mucocutaneous | No |
| 145 | F | SAPS \| SLE | 49 \| 1998 | 19 | No | No | No | W | W | W | 2 - 3 | - | Yes | Bronchiectasis | Haemoptysis (2009) | CRNM | Mucocutaneous | No |
| 145 | F | SAPS \| SLE | 49 \| 1998 | 19 | No | No | No | W | W | W | 2 - 3 | - | Yes | Bronchiectasis | Haemoptysis (2010) | CRNM | Mucocutaneous | No |
| 145 | F | SAPS \| SLE | 49 \| 1998 | 19 | No | No | No | W | W | W | 2 - 3 | - | Yes | Bronchiectasis | Haemoptysis (2011) | CRNM | Mucocutaneous | No |
| 145 | F | SAPS \| SLE | 49 \| 1998 | 19 | No | No | No | W | W | W | 2 - 3 | - | Yes | Bronchiectasis | Haemoptysis (2012) | CRNM | Mucocutaneous | No |
| 145 | F | SAPS \| SLE | 49 \| 1998 | 19 | No | No | No | W | W | W | 2 - 3 | - | Yes | Bronchiectasis | Haemoptysis (2013) | CRNM | Mucocutaneous | No |
| 145 | F | SAPS \| SLE | 49 \| 1998 | 19 | No | No | No | W | W | W | 2 - 3 | - | Yes | Bronchiectasis | Haemoptysis (2014) | CRNM | Mucocutaneous | No |
| 145 | F | SAPS \| SLE | 49 \| 1998 | 19 | No | No | No | W | W | W | 2 - 3 | - | Yes | Bronchiectasis | Haemoptysis (2015) | CRNM | Mucocutaneous | No |
| 145 | F | SAPS \| SLE | 49 \| 1998 | 19 | No | No | No | W | W | W | 2 - 3 | - | Yes | Bronchiectasis | Haemoptysis (2017) | CRNM | Mucocutaneous | No |
| 145 | F | SAPS \| SLE | 49 \| 1998 | 19 | No | No | No | W | W | W | 2 - 3 | - | Yes | Bronchiectasis | Haemoptysis (2017) | CRNM | Mucocutaneous | No |
| 146 | F | SAPS \| SLE | 40 \| 1982 | 21 | No | ITP (unknown severity) \|- | - | W | W | W | 3 - 4 | - | Yes | Gastritis | Upper GI bleeding | CRNM | Abdominal | No |
| 147 | F | SAPS \| SLE | 44 \| 2009 | 5 | Arterial | No | No | W | W, LMWH | LMWH | 2 - 3 | - | Yes | - | GI bleeding with iron deficient anaemia (2010) | CRNM | Abdominal | No |
| 148 | M | SAPS \| SLE | 53 \| 1991 | 15 | No | ITP unknown severity (after 2000) \|- | No | AAS | W, AAS | AAS | 3 - 4 | N/A | Yes | - | Epistaxis (1997) | CRNM | Mucocutaneous | No |
| 148 | M | SAPS \| SLE | 53 \| 1991 | 15 | No | ITP unknown severity (since 2000) \|- | Yes | AAS | W AAS | AAS | 3 - 4 | N/A | Yes | - | Epistaxis (2000) | CRNM | Mucocutaneous | No |
| 148 | M | SAPS \| SLE | 53 \| 1991 | 15 | No | ITP unknown severity (since 2000) \|- | Yes | AAS | W, AAS | AAS | 3 - 4 | N/A | Yes | - | Epistaxis (2003) | CRNM | Mucocutaneous | No |
| 150 | F | SAPS \| SLE | 18 \|2009 | 7 | Venous | Mod. \| - | - | AAS | W, LMWH, AAS | AAS | 2 - 3 | N/A | Yes | - | CNS microhaemorrhages (2009) | Minor | CNS | No |
| 150 | F | SAPS \| SLE | 18 \|2009 | 7 | Venous | Mod. \| 127 | Yes | AAS | W, LMWH, AAS | AAS | 2 - 3 | N/A | Yes | - | AUB leading to anaemia requiring periodic blood transfusion (2011) | CRNM | GU | No |
| 150 | F | SAPS \| SLE | 18 \|2009 | 7 | Venous | Mod. \| 127 | Yes | AAS | W, LMWH, AAS | AAS | 2 - 3 | N/A | Yes | - | Easy bruising (2011) | Minor | Mucocutaneous | No |
| 150 | F | SAPS \| SLE | 18 \|2009 | 7 | Venous | Mod. \| 127 | Yes | AAS | W, LMWH, AAS | AAS | 2 - 3 | N/A | Yes | - | Epistaxis (2011) | Minor | Mucocutaneous | No |
| 151 | F | SAPS \| SLE | 59 \| 2007 | 11 | No | Severe \| 123 | Yes | AAS | LMWH, AAS | AAS | Standard | N/A | Yes | - | Haematuria (2015) | CRNM | GU | No |
| 153 | F | SAPS \| SLE | 17 \| 1980 | 39 | Arterial and venous | No | No | W | W | W | - | - | Yes | - | Muscular hematoma (2000) | CRNM | Soft tissue | No |
| 153 | F | SAPS \| SLE | 17 \| 1980 | 39 | Arterial and venous | No | No | W | W | W | - | - | Yes | - | Muscular hematoma (2001) | CRNM | Soft tissue | No |
| 153 | F | SAPS \| SLE | 17 \| 1980 | 39 | Arterial and venous | No | No | W | W | W | - | - | Yes | Gastritis | Upper GI bleeding | CRNM | Abdominal | No |
| 153 | F | SAPS \| SLE | 17 \| 1980 | 39 | Arterial and venous | No | No | W | W | W | - | - | Yes | - | Intra-abdominal haemorrhage (unknown origin) | CRNM | Abdominal | No |
| 153 | F | SAPS \| SLE | 17 \| 1980 | 39 | Arterial and venous | No | No | W | W | W | - | - | Yes | - | Easy bruising | Minor | Mucocutaneous | No |
| 154 | F | SAPS \| SLE | 21 \| 1980 | 29 | Venous | Severe ITP with splenectomy (before 2004) \| - | Yes | W | W | W | - | - | Yes | - | Muscular hematoma (1995) | CRNM | Soft tissue | No |
| 158 | F | SAPS \| SLE | 41 \| 2015 | 4 | No | Severe ITP with splenectomy (1990) \| > 300 (since 2015) | No | RVX | W, RVX | W, RVX | 2 - 3 | - | Yes | - | AUB (2017) | Minor | GU | No |
| 158 | F | SAPS \| SLE | 41 \| 2015 | 4 | No | Severe ITP with splenectomy (1990) \| > 300 (since 2015) | No | - | W, RVX | W, RVX | 2 - 3 | - | Yes | - | Muscular hematoma | CRNM | Soft tissue | No |
| 158 | F | SAPS \| SLE | 41 \| 2015 | 4 | No | Severe ITP with splenectomy (1990) \| > 300 (since 2015) | No | - | W, RVX | W, RVX | 2 - 3 | - | Yes | - | Easy bruising | Minor | Mucocutaneous | No |
| 168 | F | SAPS \| SLE | 20 \| 2009 | 10 | Arterial | No | No | LMWH, AAS | W, LMWH, AAS | LMWH, AAS | 3 - 4 | - | Yes | - | CNS microhaemorrhages (2018) | Minor | CNS | No |
| 169 | F | SAPS \| SLE | 22 \| 2010 | 9 | No | No | No | W | W | W | 3 - 4 | - | Yes | - | AUB leading to iron deficient anaemia (2012) | CRNM | GU | No |
| 169 | F | SAPS \| SLE | 22 \| 2010 | 9 | No | No | No | W | W | W | 3 - 4 | - | Yes | - | Gum bleeding (2019) | Minor | Mucocutaneous | No |
| 170 | F | SAPS \| SLE | 56 \| 2007 | 12 | No | No | No | RVX | W, RVX | RVX | 2 - 3 | 20mg | Yes | - | CNS microhaemorrhages (2019) | Minor | CNS | No |
| 176 | F | SAPS \| SLE | 50 \| 2009 | 10 | Venous | No | No | W | W | W | 2 - 3 | - | Yes | - | CNS microhaemorrhages (2013) | Minor | CNS | No |
| 179 | F | SAPS \| SLE | 31 \| 1996 | 8 | Arterial | No | No | W | W | W | 2.5 - 3.5 | - | Yes | Duodenitis | Upper GI bleeding (2016) | CRNM | Abdominal | No |
| 179 | F | SAPS \| SLE | 31 \| 1996 | 8 | Arterial | No | No | W | W | W | 2.5 - 3.5 | - | Yes | - | CNS microhaemorrhages (2016) | Minor | CNS | No |
| 181 | F | SAPS \| SLE | 43 \| 2014 | 5 | Arterial | No | No | - | W, LMWH | LMWH | Standard | Standard | Yes | - | CNS microhaemorrhages (2016) | Minor | CNS | No |
| 182 | F | SAPS \| SLE | 25 \| 2001 | 18 | Arterial, venous, and microvascular | Mod. \| 54 | Yes | - | W, LMHW | LMWH | 2.5 | - | Yes | - | CNS microhaemorrhages (2018) | Minor | CNS | No |
| 183 | F | SAPS \| SLE | 39 \| 2004 | 15 | Arterial | No | No | W | W | W | 2.5 - 3.5 | - | Yes | - | AUB (2014) | Minor | GU | No |
| 184 | F | SAPS \| SLE | 26 \| 2012 | 7 | No | Mod. \| - | No | W | W, AAS | W, AAS | 3 - 4 | - | Yes | - | AUB (2013) | CRNM | GU | No |
| 188 | F | SAPS \| SLE | 22 \| 2014 | 5 | No | No | No | RVX | RVX | RVX | - | - | Yes | - | AUB (2018) | CRNM | GU | No |
| 189 | F | SAPS \| SLE | 21 \| 2005 | 14 | Arterial | No | No | W, AAS | W, AAS | W, AAS | 3 - 4 | - | Yes | - | Easy bruising (2019) | Minor | Mucocutaneous | No |
| 189 | F | SAPS \| SLE | 21 \| 2005 | 14 | Arterial | No | No | W, AAS | W, AAS | W, AAS | 3 - 4 | - | Yes | - | Epistaxis (2019) | Minor | Mucocutaneous | No |
| 190 | F | SAPS \| SLE | 27 \| 2018 | 1 | No | ITP (unknown severity) \| - | - | W | W | W | 2 - 3 | - | Yes | - | CNS microhaemorrhages (2019) | Minor | CNS | No |
| 191 | F | SAPS \| SLE | 16 \| 2004 | 15 | No | No | No | W, AAS | W, AAS | W, AAS | 2 - 3 | - | Yes | - | AUB (2014) | Minor | GU | No |
| 191 | F | SAPS \| SLE | 16 \| 2004 | 15 | No | No | No | W, AAS | W, AAS | W, AAS | 2 - 3 | - | Yes | - | Epistaxis (2015) | CRNM | Mucocutaneous | No |
| 191 | F | SAPS \| SLE | 16 \| 2004 | 15 | No | No | No | W, AAS | W, AAS | W, AAS | 2 - 3 | - | Yes | - | Epistaxis (2017) | CRNM | Mucocutaneous | No |
| 193 | F | SAPS \| SLE | 20 \| 2004 | 15 | Arterial | No | No | W | W | W | 2.5 - 3.5 | - | Yes | - | AUB (2019) | Minor | GU | No |
| 204 | F | SAPS \| SLE | 42 \| 1997 | 22 | No | No | No | W, Clopi | W, Clopi | W, Clopi | 2 - 3 | - | Yes | Intestinal angiodysplasia | Lower GI bleeding leading to iron deficient anaemia (2004) | CRNM | Abdominal | No |
| 206 | F | SAPS \| RA | 50 \| 2010 | 9 | No | No | No | W | W | W | 2.5 - 3.5 | - | Yes | - | CNS microhaemorrhage (2018) | Minor | CNS | No |
| 211 | M | SAPS \| UCTD | 53 \| 2000 | 19 | No | Mild \| - | - | W | W, LMWH, AAS | LMWH | - | - | Yes | - | Easy bruising (2003) | CRNM | Mucocutaneous | No |
| 211 | M | SAPS \| UCTD | 53 \| 2000 | 19 | No | Mild \| 160 | No | LMWH | W, LMWH, AAS | LMWH | - | - | Yes | - | Muscular hematoma (2017) | CRNM | Soft tissue | No |
| 211 | M | SAPS \| UCTD | 53 \| 2000 | 19 | No | Mild \| 154 | No | LMWH | W, LMWH, AAS | LMWH | - | - | Yes | - | Muscular hematoma (2019) | CRNM | Soft tissue | No |

Data are shown when available. Each row represents a distinct non-major-BE (minor-BE, **n=55**; CRNM-BE, **n=74**) affecting **67** thrombotic APS patients regardless of whether they were also affected by major-BE (**Supplementary Table 2**).

* Recurrent thrombotic events during antithrombotic therapy, regardless of the treatment modality and/or the intensity of anticoagulation; † Thrombocytopenia severity is stated as the lowest value ever present; ‡ Mild: 100x10^9^/L to 150x10^9^/L, Mod.: 50x10^9^/L to 100x10^9^/L, Severe: <50x10^9^/L; § Near bleeding event. ¶ Last treatment modality; ** Latest INR target value/aimed anticoagulation intensity. AAS, acetylsalicylic acid; AUB, abnormal uterine bleeding; Clopi, clopidogrel; CNS, central nervous system; CRNM, clinically relevant non-major-BE; F, female; FPNX, fondaparinux; GI, gastrointestinal; GU, genitourinary; ICU, intensive care unit; INR, international normalized ratio; ITP, immune thrombocytopenia purpura; LMWH, low molecular weight heparin; M, male; N/A, not applicable; PAPS, primary antiphospholipid syndrome; PTL, platelets; RVX, rivaroxaban; RA, rheumatoid arthritis; SAPS, secondary antiphospholipid syndrome; SLE, systemic lupus erythematosus; UCTD, undifferentiated connective tissue disease; W, warfarin.
